# A New Step in the Optimization of the Chambadal Model of the Carnot Engine

**DOI:** 10.3390/e24010084

**Published:** 2022-01-04

**Authors:** Michel Feidt, Monica Costea

**Affiliations:** 1 Laboratory of Energetics, Theoretical and Applied Mechanics (LEMTA), URA CNRS 7563, University of Lorraine, 54518 Vandoeuvre-lès-Nancy, France; michel.feidt@univ-lorraine.fr; 2 Department of Engineering Thermodynamics, University Politehnica of Bucharest, 060042 Bucharest, Romania

**Keywords:** optimization, Carnot engine, Chambadal model, entropy production action, efficiency at maximum power

## Abstract

This paper presents a new step in the optimization of the Chambadal model of the Carnot engine. It allows a sequential optimization of a model with internal irreversibilities. The optimization is performed successively with respect to various objectives (e.g., energy, efficiency, or power when introducing the duration of the cycle). New complementary results are reported, generalizing those recently published in the literature. In addition, the new concept of entropy production action is proposed. This concept induces new optimums concerning energy and power in the presence of internal irreversibilities inversely proportional to the cycle or transformation durations. This promising approach is related to applications but also to fundamental aspects.

## 1. Introduction

Sadi Carnot had a crucial contribution to thermostatics that designated him as a co-founding researcher of equilibrium thermodynamics. He has shown that the efficiency of a thermo-mechanical engine is bounded by the Carnot efficiency *η_C_* [1]. Assuming an isothermal source at *T_HS_*, and an isothermal sink at *T_CS_* < *T_HS_*, and in between the cycle composed by two isothermals in perfect thermal contact with the source and sink, and two isentropics, he obtained:

(1) $$\eta_{C}=1-\frac{T_{CS}}{T_{HS}}.$$

Since that time, many papers have used the keyword “Carnot engine” (1290 papers from Web of Science on 17 September 2021). That same day on Web of Science, we noted 104 papers related to the keyword “Carnot efficiency”.

Among these papers, some are related to the connection between energy, efficiency, and power optimization. The most cited paper is probably that of Curzon and Ahlborn [2,3]. These authors proposed in 1975 an expression of the efficiency according to the first law of thermodynamics $\eta_{I}\left(MaxW\right)$ at the maximum mechanical energy and at the maximum power $\dot{W}$ for the endo-reversible configuration of the Carnot cycle (no internal irreversibility for the converter in contact with two isothermal heat reservoirs):

(2) $$\eta_{I,endo}\left(MaxW\right)=1-\sqrt{\frac{T_{CS}}{T_{HS}}}$$

This result is well-known as the *nice radical*, and it has been recently reconsidered in the previous Special Issue *Carnot Cycle and Heat Engine Fundamentals and Applications I* [3] and particularly in [4]. This last paper reports on the progress in Carnot and Chambadal modeling of thermomechanical engines by considering entropy production and heat transfer entropy in the adiabatic case (without heat losses).

The proposed paper gives back the basis of the modeling and a summary of the main results obtained recently for an endo-irreversible Carnot engine. Furthermore, the performance analysis of an extended Chambadal configuration is considered by including the converter irreversibilities. Emphasis is placed on the entropy production method, which is preferred over the ratio method.

## 2. Summary of Obtained Results for Carnot Endo-Irreversible Configuration

The consideration of *endo-irreversible* Carnot engine modeling was recently developed [5]. The approach considering as a reference the heat transfer entropy released at the sink Δ*S_S_* (maximum entropy available at the source in the reversible case) [5] confirmed that the work per cycle results (see Appendix A):

(3) $$W=\left(T_{HS}–T_{CS}\right)\left(\Delta S_{S}–\Delta S_{I}\right),\;$$

where Δ*S_I_* is the entropy production due to the internal irreversibilities of the cycle throughout the four thermodynamic transformations (two adiabatic and two isothermal ones).

For an engine without thermal losses, the following expression of the thermal efficiency was retrieved:

(4) $$\eta_{I}=\eta_{C}\left(1-d_{I}\right),$$

where $d_{I}=\frac{\Delta S_{I}}{\Delta S_{S}}$ is a coefficient of the converter’s internal irreversibility during the cycle.

This parameter was introduced by Novikov [6] and Ibrahim et al. [7] in slightly different forms.

The reversible limit (*d_I_* = 0) in Equation (4) restores the Carnot cycle efficiency associated with equilibrium thermodynamics.

Since the reversibility is unattainable, it appears that the optimization (maximization) of the mechanical energy at the given parameters (Δ*S_S_*, *T_HS_*, and *T_CS_*) is related to the minimization of the entropy production (as was proposed by Gouy [8]).

The assumption that each of the four transformations of the endo-irreversible cycle takes place with a duration *τ_i_* (*i* = 1–4), leading to the inverse proportionality to *τ_i_* of the corresponding entropy production:

(5) $$\Delta S_{Ii}=\frac{C_{Ii}}{\tau_{i}},$$

where *C_Ii_* represents the irreversibility coefficients, whose unit is Js/K [5].

These coefficients are *irreversible entropic actions* by analogy to the energy (mechanical) action (Js).

By performing cycle energy optimization using the Lagrange multipliers method with the constraint of the cycle’s finite time duration *τ,* one obtains the maximum work per cycle [5]:

(6) $$Max_{1}W=W_{endo}-\frac{\Delta T_{S\;}}{\tau }{\left(\sum_{i}\sqrt{C_{Ii}}\right)}^{2},$$

where Δ*T_S_* = *T_HS_* − *T_CS_*.

The efficiency at the maximum finite time work becomes

(7) $$\eta_{I}\left(Max_{1}W\right)=\eta_{C}\left(1–\frac{{\left(\sum_{i}\sqrt{C_{Ii}}\right)}^{2}}{\tau \cdot \Delta S_{S}}\right),$$

where $\tau \Delta S_{S}$ is the available entropic transfer action of the cycle.

The new result provided by Equation (7) gives back the Carnot efficiency limit for the reversible case (*C_Ii_* = 0). These calculations have been pursued for the case of power optimization, where Δ*S_S_*, *T_HS_*, and *T_CS_* remain parameters. It was shown that a value of the cycle duration $\tau^{*}$ corresponding to $Max\bar{\dot{W}}$, the mean power output over the cycle, exists, and it is expressed as

(8) $$\tau^{*}=8\frac{C_{Ii}}{\Delta S_{S}},$$

and

(9) $$Max\bar{\dot{W}}=\frac{\Delta T_{S}\cdot \Delta S_{S}{}^{2}}{16\;C_{Ii}}.\;$$

Equation (9) proves that $Max\bar{\dot{W}}$ is a decreasing function of the total entropic action of the cycle and that the associated efficiency with the maximum of the mean power corresponds to half the Carnot efficiency, as appeared repeatedly in some recent works [9,10,11].

## 3. Summary of the Obtained Results for the Chambadal Configuration

**Figure 1:**
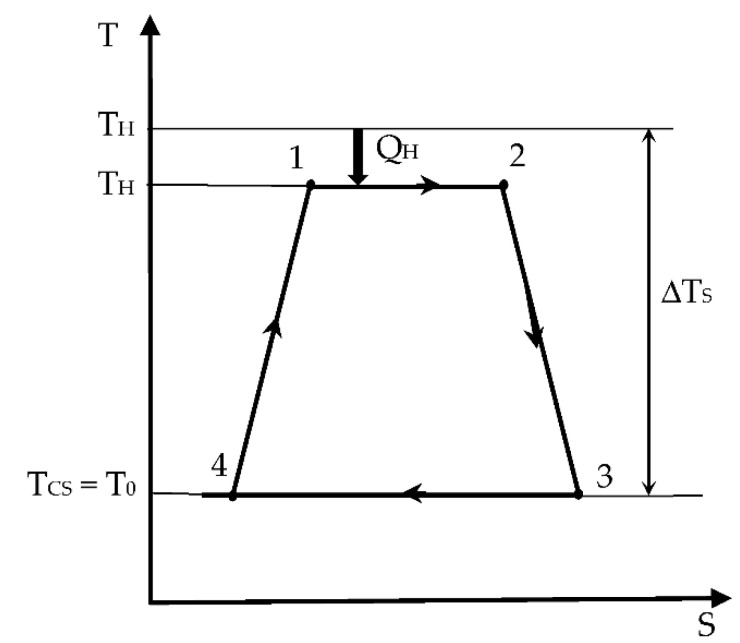
Representation of the associated cycle with the Chambadal engine in a *T*-*S* diagram.

In the present paper, we intend to reconsider the approach of the Chambadal model of a Carnot engine [12]. This configuration is common for thermomechanical engines, since the cold sink mainly refers to the environment (i.e., the atmosphere or water sink). This corresponds to the Chambadal approach (Figure 1), with a temperature gradient at the hot source (*T_HS_*, *T_H_*) but with perfect thermal contact at the sink (*T_CS_* or *T*_0_ at ambient temperature).

We propose here to extend the results (Equations (6)–(9)) to enhance the Chambadal configuration modeling. This extension starts from the endo-irreversible case, to which external irreversibilities due to heat transfer between the hot finite source and the converter are added. Thus, the new results obtained complete the endo-irreversible Carnot model [5] and an earlier paper on Chambadal configuration [12].

### 3.1. The Modified Chambadal Engine

To help understand the extension of the modeling in Section 3, we report here the case with the following hypothesis:

1. *Adiabaticity* (no thermal losses);
2. *Linear heat transfer law* at the source such that
  (10) $$Q_{H}=G_{H}\left(T_{HS}–T_{H}\right),$$
  where *G_H_* is the heat transfer conductance expressed by $G_{H}=K_{H}\tau$ when we consider the mean value over the cycle duration *τ* or $G_{H}=K_{H}^{\prime }\tau_{H}$ when we consider the mean value over the isothermal heat transfer at the hot source (as was performed by Curzon and Ahlborn [2]).

Equation (10) corresponds to the heat expense of the engine.

Note that other heat transfer laws, namely the Stefan–Boltzmann radiation law, the Dulong–Petit law, and another phenomenological heat transfer law can be considered in the maximum power regime search [13];

3. *Presence of irreversibility* in the converter (internal irreversibility).

Two approaches are proposed in the literature, which introduce the internal irreversibility of the engine by (1) the *irreversibility ratio I_H_*, [6,7], respectively (2) the *entropy production over the cycle* Δ*S_I_*, [5]. 

We preconized this second approach for a long time. We also note that the original model of Chambadal is endo-reversible [14]. Hence, we prefer to name the present model the “modified Chambadal model” due to some other differences that will be specified hereafter.

Note that only the second approach regarding the presence of irreversibilities in the converter will be considered in the following section.

### 3.2. Optimization of the Work per Cycle of the Modified Chambadal Engine with the Entropy Production Method

The first law of thermodynamics applied to the cycle implies conservation of energy, written as

(11) $$W=Q_{conv}-Q_{S}$$

where $Q_{conv}$ and $Q_{S}$ are defined in Appendix A.

One supposes here that Δ*S_I_* is a parameter representing the total production of entropy over the cycle composed by four irreversible transformations. Thus, the entropy balance corresponds to

(12) $$\frac{Q_{conv}}{T_{H}}+\Delta S_{I}=\frac{Q_{S}}{T_{0}}.$$

By combining Equations (11) and (12), we easily obtained

(13) $$W=Q_{conv}\left(1-\frac{T_{0}}{T_{H}}\right)-T_{0}\Delta S_{I}.$$

If $Q_{conv}$ (Δ*S_conv_*) is a given parameter, we retrieve the Gouy-Stodola theorem stating that *Max W* corresponds to min Δ*S_I_* with the known consequences reported in Section 4.1.

### 3.3. Optimization of the Work per Cycle of the Modified Chambadal Engine with the Heat Transfer Constraint

In this case, the energy balance between the source and isothermal transformation implies the combination of Equations (13) and (A1):

(14) $$W=\left(Q_{H}-T_{H}\Delta S_{IH}\right)\left(1-\frac{T_{0}}{T_{H}}\right)-T_{0}\Delta S_{I}.$$

Knowing *Q_H_* from Equation (10), one obtains

(15) $$W=G_{H}\left(T_{HS}–T_{H}\right)\left(1-\frac{T_{0}}{T_{H}}\right)-T_{H}\Delta S_{IH}-T_{0}\Delta S_{I}^{\prime },$$

where $\Delta S_{I}^{\prime }=\Delta S_{IEx}+\Delta S_{IC}+\Delta S_{ICo}$.

The maximum of *W* with respect to *T_H_* is obtained for

(16) $$T_{H}^{*}=\sqrt{\frac{T_{HS}T_{0}}{1+s_{I}}},$$

where $s_{I}=\frac{\Delta S_{IH}}{G_{H}}$, a specific ratio relative to the irreversible isothermal transformation *T_H_*.

Finally, the expression of $Max_{1}W$ yields

(17) $$Max_{1}W=G_{H}{\left(\sqrt{T_{HS}}-\sqrt{\left(1+s_{I}\right)T_{0}}\right)}^{2}-T_{0}\Delta S_{I}.$$

## 4. Complement to the Previous Results

Now, we will consider the time variable related to entropy production for each thermodynamic transformation, defined as $\Delta S_{Ii}=\frac{C_{Ii}}{\tau_{i}}$. This form of the entropy production satisfying the second law induces that the entropy production method is well adapted to subsequent optimizations of energy and power as well.

### 4.1. Work Optimization Relative to the Time Variables

The expression of $Max_{1}W$ with *G_H_* as an extensive parameter (Equation (17)) shows that $Max_{1}W$ is always the optimum in the endo-reversible case. Nevertheless, if there are separate irreversibilities for each cycle transformation (as is the case with finite entropic actions), the irreversibility on the high temperature isotherm possesses a specific role (see Equation (17) and the $s_{I}$ ratio).

The constraint on the transformation duration or preferably frequencies *f_i_* (finite cycle duration) allows one to seek for the optimal transformation duration allocation (see Appendix B for the derivation).

We obtained $Max_{2}W$ for the following optimal durations:

(18) $$\tau_{H}{}^{*}=\sqrt{\sqrt{T_{0}T_{HS}}\frac{C_{IH}}{\lambda }}\;,$$

and

(19) $$\tau_{i}{}^{*}=\sqrt{\frac{T_{0}C_{Ii}}{\lambda }}\;,\;$$

where *λ* is given in Appendix B and *i* = *Ex*, *C*, *Co.*

Thus, the second optimization of *W* (see Appendix B) leads to

(20) $$Max_{2}W\approx W_{endo}-\frac{T_{0\;}}{\tau }N^{2}\;.\;$$

Furthermore, a third sequential optimization could be performed by considering the finite entropic action as a new constraint. This case is not developed here for brevity reasons.

### 4.2. Power Optimization in the Case of a Finite Heat Source (When G_H_ Is the Parameter)

The mean power of the modified Chambadal cycle for the condition of maximum work $Max_{2}W$ is defined by

(21) $$\bar{\dot{W}}\left(Max_{2}W\right)=\frac{W_{endo}}{\tau }–\frac{T_{0\;}}{\tau^{2}}N^{2},\;$$

where $W_{endo}=G_{H}{\left(\sqrt{T_{HS\;}}–\sqrt{T_{0\;}}\right)}^{2}$ is the mechanical work output of the endo-reversible engine.

The power is maximized with respect to the cycle period *τ*. Thus, the expression of the optimum period is

(22) $$\tau^{*}=\frac{2T_{0}N^{2}}{W_{endo}}\;.\;$$

This expression is analogous to the similar results obtained in [5], leading to

(23) $$Max\bar{\dot{W}}=\frac{W_{endo}{}^{2}}{4T_{0}N^{2}}\;.\;$$

The action of entropy production appearing in *N* diminishes the mean power of the engine. At the given endo-reversible work, the maximum power corresponds to the minimum of the *N* function, depending on the four entropy actions of the cycle, such that

(24) $$N=\sqrt{\frac{T_{0}}{T_{HS}}C_{IH}}+\sqrt{C_{IEx}}+\sqrt{C_{IC}}+\sqrt{C_{ICo}}\;.\;$$

The main difference between Equation (23) and the previous results [5] comes from the imperfect heat transfer between the source and the converter in the Chambadal model.

## 5. Discussion

This paper proposed that the Special Issue *Carnot Cycle and Heat Engine Fundamentals and Applications II* completes the previous paper [12] published in Special Issue 1 and adds new results to a recently published paper [5].

Whatever variable is chosen for the modified Chambadal model work optimization (*T_H_* or Δ*S*), the same optimum for the work per cycle is obtained with parameters *G_H_*, *T_HS_*, and *T*_0_.

It appears that by introducing the duration of each transformation *τ_i_* and the period of the cycle *τ*, the modified Chambadal model satisfies the Gouy-Stodola theorem. At the minimum of entropy production, the optimal durations are dependent on the transformation entropy actions. This result is new to our knowledge.

This new concept [5] allows a new subsequent sequential optimization. The optimal allocation of the entropy action coefficients is slightly different from the equipartition (a new form of the equipartition theorem [15,16]).

Thus, the fundamental aspect related to irreversibilities through the *new concept of entropy production action* seems promising. Furthermore, this new concept could contribute to the improvement of the global system analysis by conducting optimal dimension allocation. In this respect, finite physical dimensions analysis could be a complementary way to correlate with exergy analysis.

Further extensions of this work are foreseen in the near future.

## 6. Conclusions

Similarities and differences present in the literature regarding the optimization of energy, first law efficiency, and power of the modified Chambadal engine have been resituated and summarized since the publication of [12].

This approach allows for highlighting the evolution of the obtained results from the reversible Carnot engine case (thermostatics) to the endo-irreversible models related to the approaches of Novikov [6] and Ibrahim et al. [7] or to the entropy production method that we promote.

By generalizing a proposal from Esposito et al. [9] and defining the new concept of entropic action through a coefficient *C_I_* (Js/K) for the entropy production of transformations all along the cycle, we achieved a new form of power optimization different from the one of Curzon and Ahlborn, since the internal converter irreversibilities and the heat transfer irreversibility between the heat source and converter were accounted for.

The maximum work per cycle was obtained for the irreversible cycle case. It depended on the entropic action coefficient of the four transformations of the cycle *C_Ii_*, after which the power of the engine was sequentially optimized.

An optimal period of the cycle τ* appeared, corresponding to the maximum mean power of the cycle. It generalized the recent published results [5] for a modified Chambadal engine.

This research continues to be developed by our team to obtain more general results.

## Figures and Tables

**Figure A1:**
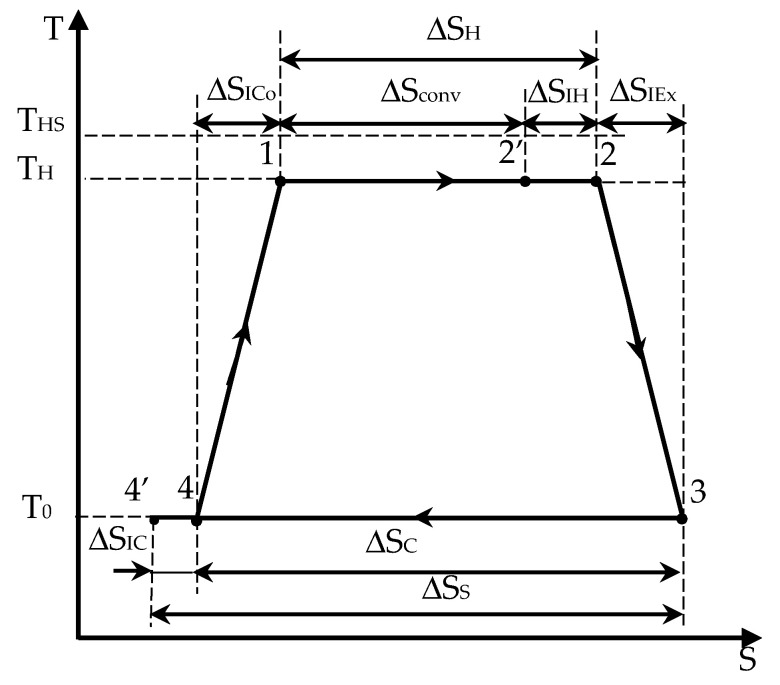
Carnot engine cycle with internal irreversibilities along the four transformations of the cycle, illustrated in a *T*-*S* diagram.

## Appendix A. Work per Cycle of the Modified Chambadal Engine with the Entropy Production Method

It results from Figure A1 that the various heats exchanged over the irreversible cycle (1–2–3–4) are expressed as follows:

- $Q_{H}=T_{H}\Delta S_{H}$ is the heat received by the cycled medium from the hot source (energy expense), corresponding to the heat transfer at the hot side;
- $Q_{conv}=T_{H}\Delta S_{conv}$, heat converted in mechanical energy during the isothermal process at $T_{H}$, with corresponding production of entropy $\Delta S_{IH}$ such that:
  (A1) $$Q_{conv}=T_{H}\left(\Delta S_{H}-\Delta S_{IH}\right).\;$$
- $Q_{C}=T_{0}\Delta S_{C}$, where $\Delta S_{C}=\Delta S_{S}-\Delta S_{IC}$.

Note that $\Delta S_{IC}$ is the entropy production during the irreversible isotherm at *T*_0_ and $\Delta S_{S}$ is the entropy rejected to the sink such that $Q_{S}=T_{0}\Delta S_{S}$.

Thus, the entropy balance over the cycle is

(A2) $$\Delta S_{conv}+\Delta S_{I}=\Delta S_{S}\;$$

The total entropy production over the cycle $\Delta S_{I}$ is represented by

(A3) $$\Delta S_{I}=\Delta S_{IH}+\Delta S_{IEx}+\Delta S_{IC}+\Delta S_{ICo},\;$$

where

Δ*S_IH_* is the entropy production during the isothermal transformation at *T_H_*, Δ*S_IE_* is the entropy production during the adiabatic expansion from *T_H_* to *T*_0_, Δ*S_IC_* is the entropy production during the isothermal transformation at *T*_0_, and Δ*S_ICo_,* is the entropy production during the adiabatic compression from *T*_0_ to *T_H_*.

The energy balance over the cycle for the system comprising the converter, the heat source, and the sink (with the source and sink as perfect thermostats) provides

(A4) $$W=Q_{conv}-Q_{S}.\;$$

Various forms of mechanical energy are obtainable from this point by combining the preceding relations. Thus, one may express *W* as follows:

1. With $\Delta S_{conv}$ as the reference entropy:
  (A5) $$W=T_{H}\Delta S_{conv}-T_{0}\Delta S_{S},\;$$
  (A6) $$W=\left(T_{H}-T_{0}\right)\Delta S_{conv}-T_{0}\Delta S_{I}.$$
2. With $\Delta S_{S}$ as the reference entropy:
  (A7) $$W=\left(T_{H}-T_{0}\right)\Delta S_{S}-T_{H}\Delta S_{I}.$$
3. With $\Delta S_{S}$ or $\Delta S_{S}$ as the reference entropy:
  (A8) $$W=T_{H}\left(\Delta S_{H}-\Delta S_{IH}\right)-T_{C}\left(\Delta S_{C}+\Delta S_{IC}\right).$$

We prefer to choose between Equations (A6) and (A7). Note that Equation (A7) was the one used by Esposito et al. [9].

We use Equation (A6) here because it gave back known results, particularly the Gouy-Stodola theorem, with $\Delta S_{conv}$ being a parameter. Thus, the maximum energy occurs when $\Delta S_{I}=0$ such that

(A9) $$W_{endo}=\left(T_{H}-T_{0}\right)\Delta S_{conv}.\;$$

This corresponds to the endo-reversible model of Chambadal.

In Section 3, we proposed a complete Chambadal model taking account entropy production all along the cycle.

## Appendix B. Work Optimization Relative to Time (Frequency)

Using the Lagrange multipliers method with the frequencies $f_{i}=\frac{1}{\tau_{i}}$ as variables, we get the following function:

(A10) $$\begin{array}{c} L\left(f_{i}\right)={\left(\sqrt{G_{H}T_{HS\;}}-\sqrt{\left(G_{H}+C_{IH}f_{H}\right)T_{0}}\right)}^{2}\\ \;\;\;\;\;\;\;\;\;\;\;\;\;\;\;-T_{0}\left(C_{IH}f_{H}+C_{IEx}f_{Ex}+C_{IC}f_{C}+C_{ICo}f_{Co}\right)\\ \;\;\;\;\;\;\;\;\;\;\;\;\;\;\;-\lambda (\frac{1}{f_{H}}+\frac{1}{f_{Ex}}+\frac{1}{f_{C}}+\frac{1}{f_{Co}}-\tau ).\; \end{array}$$

The vector of optimal values is

(A11) $$f_{Ex}^{*}=\sqrt{\frac{\lambda }{T_{0}C_{IEx}}}\;\;\;\;\;\;\;;\;\;\;\;\;\;\;\;f_{C}^{*}=\sqrt{\frac{\lambda }{T_{0}C_{IC}}}\;\;\;\;\;\;\;\;\;;\;\;\;\;\;\;\;\;f_{Co}^{*}=\sqrt{\frac{\lambda }{T_{0}C_{ICo}}},\;$$

Additionally, the following is a non-linear equation to solve numerically for $f_{H}^{*}$:

(A12) $$f_{H}^{2}=\lambda \sqrt{\frac{G_{H}+C_{IH}f_{H}}{G_{H}}}\frac{1}{\sqrt{T_{HS}T_{0}}C_{IH}}.$$

In the reasonable case of low irreversibility on the *T_H_* isotherm $\left(C_{IH}f_{H}\ll G_{H}\right)$, a good approximation of $f_{H}^{*}$ is

(A13) $$f_{H}^{*}=\sqrt{\frac{\lambda }{\sqrt{T_{HS}T_{0}}C_{IH}}}.$$

The finitude constraint on $\tau_{i}$ allows for determining the $\sqrt{\lambda }$ expression as

(A14) $$\sqrt{\lambda }=\frac{N\sqrt{T_{0}}}{\tau },$$

where

(A15) $$N=\sqrt{\frac{T_{0\;}}{T_{HS}}C_{IH}}+\sqrt{C_{IEx}}+\sqrt{C_{IC}}+\sqrt{C_{ICo}}\;.\;$$

Finally, we get

(A16) $$Max_{2}W\approx W_{endo}-\frac{T_{0\;}}{\tau }N^{2}\;.$$
